# Long-Range Temporal Correlations in Resting State Beta Oscillations are Reduced in Schizophrenia

**DOI:** 10.3389/fpsyt.2019.00517

**Published:** 2019-07-19

**Authors:** James K. Moran, Georgios Michail, Andreas Heinz, Julian Keil, Daniel Senkowski

**Affiliations:** ^1^Department of Psychiatry and Psychotherapy, St. Hedwig Hospital, Charité-Universitätsmedizin Berlin, Berlin, Germany; ^2^Biological Psychology, Christian-Albrechts University Kiel, Kiel, Germany

**Keywords:** schizophrenia, resting-state EEG, alpha oscillations, beta oscillations, long-range temporal correlations (LRTC)

## Abstract

Symptoms of schizophrenia (SCZ) are likely to be generated by genetically mediated synaptic dysfunction, which contribute to large-scale functional neural dysconnectivity. Recent electrophysiological studies suggest that this dysconnectivity is present not only at a spatial level but also at a temporal level, operationalized as long-range temporal correlations (LRTCs). Previous research suggests that alpha and beta frequency bands have weaker temporal stability in people with SCZ. This study sought to replicate these findings with high-density electroencephalography (EEG), enabling a spatially more accurate analysis of LRTC differences, and to test associations with characteristic SCZ symptoms and cognitive deficits. A 128-channel EEG was used to record eyes-open resting state brain activity of 23 people with SCZ and 24 matched healthy controls (HCs). LRTCs were derived for alpha (8–12 Hz) and beta (13–25 Hz) frequency bands. As an exploratory analysis, LRTC was source projected using sLoreta. People with SCZ showed an area of significantly reduced beta-band LRTC compared with HCs over bilateral posterior regions. There were no between-group differences in alpha-band activity. Individual symptoms of SCZ were not related to LRTC values nor were cognitive deficits. The study confirms that people with SCZ have reduced temporal stability in the beta frequency band. The absence of group differences in the alpha band may be attributed to the fact that people had, in contrast to previous studies, their eyes open in the current study. Taken together, our study confirms the utility of LRTC as a marker of network instability in people with SCZ and provides a novel empirical perspective for future examinations of network dysfunction salience in SCZ research.

## Introduction

Many people with schizophrenia (SCZ) suffer from delusions and hallucinations as well as deficits in cognitive function across the lifespan. These symptoms have been linked to dysfunction in oscillatory activity of neuronal populations in the brain (1). Oscillatory activity in individual brain areas of SCZ patients has been shown to be noisier and connected with worse performance in cognitive tasks (2–4), as well as unisensory (5, 6) and multisensory stimulus processing (7–9). Moreover, resting state electrophysiological studies have shown abnormalities in resting state power (10–12) and spatial connectivity [see Ref. (13) for review]. The *dysconnection hypothesis* (14, 15) posits that dysfunctional interplay between and across neural assemblies of SCZ patients results from a combination of genetic abnormalities, compounded by failures in developmental tuning of synapses. Since oscillations are thought to play a role in the precise temporal relationships necessary for neural responses, abnormal oscillations in neuronal assemblies likely play a central part in the complex etiology of SCZ (1, 15).

It is possible that the dysconnection in functional oscillatory activity in SCZ also has a temporal dimension. Temporal autocorrelations of the power in oscillatory bands have been shown to persist across different time windows. This is called a long-range temporal correlation (LRTC) (16). The stability of cortical neuronal networks is maintained by the coordinated firing of GABA ergic and glutamatergic neuronal assemblies (1). The putative dysfunction in these nuerotransmitter systems in people with SCZ is hypothesized to have a genetic component, which is thought to underlie temporal instability in oscillatory power. The various negative and positive symptoms of SCZ could be partly the result of this instability (17). In healthy individuals, LRTC reflects a balance between stability and flexibility in neuronal assemblies (18). An imbalance in this stability and flexibility likely plays an important role in various psychiatric and neurological disorders. For example, in epilepsy, LRTC is stronger in seizure-affected areas (19). In contrast, early-stage Alzheimer’s disease shows weakened LRTC (20). The psychopathology of SCZ could similarly be a reflection of instability in neural networks, as expressed through diminished LRTC—i.e., more random-like fluctuations—in the temporal dynamics of neuronal oscillations.

Previously, Nikulin et al. (21) compared LRTC in people with SCZ and healthy controls (HCs) in a resting state electroencephalography (EEG) study, where individuals had their eyes closed. The authors recorded EEG data from 21 scalp electrodes and examined LRTC for different frequency bands. People with SCZ compared with HCs showed reduced LRTC in alpha (8-12 Hz) and beta 16-24 Hz oscillatory bands.

Alpha and beta-band activities have been frequently related to top-down processing. Alpha-band activity plays a role in top-down processing of sensory stimuli (22), as well as more generally focusing attention *via* inhibition of irrelevant stimuli, especially in visual regions (23). Beta-band activity is related to maintenance of motor control and cognitive processes (24). Kopell et al. (25) have suggested that beta-band activity is also crucial in providing a memory for cortical network activity. Whereas gamma circuits originating in superficial cortical layers are transient and stimulus-dependent, beta-band activity from deeper cortical layers organizes and sustains gamma networks across longer periods. This is relevant to SCZ, as diminished gamma oscillatory power is frequently observed in perceptual tasks in this patient group (1).

Nikulin et al. (21) could reasonably speculate that the beta differences observed in their study originated in frontal and motor regions; however, the low spatial resolution of their 21 electrode EEG setup precluded any test of this. Furthermore, the adapted eyes-closed measure employed by Nikulin et al. (21) could add some aspects of attentional bias, since eyes closed can easily promote early stages of sleep (26, 27). Finally, other researchers have found parallels in results for LRTC in depression to different individual symptoms (28, 29). A similar consideration of positive and negative symptoms of SCZ, as well as cognitive capacity could also further specify the function of any LRTC group differences.

The present study aims to build on the study of Nikulin et al. (21) by testing LRTC in people with SCZ and HCs using high-density EEG recordings from 128 scalp electrodes cluster-based permutation tests, and recording data while individuals had their eyes open. In addition, for exploratory purposes, data were source projected, providing first insights into the cortical regions showing diminished LRTC during rest in SCZ. Due to a lack of sufficient data quality and other factors, a more rigorous source-level LRTC analysis was beyond the scope of the current study. We also related LRTC to dimensional aspects of SCZ symptomatology, together with tests of cognitive deficits. This serves the purpose of replicating the results of Nikulin et al. (21) in an independent sample. There is a generally acknowledged need for replication in neuroscience, with its large multivariate outcome measures (30). This is particularly so in resting-state studies of clinical patients, which are unconstrained by specific experimental stimuli, and where patient symptom profiles are heterogeneous.

## Methods

### Sample and Clinical Data

Twenty-three people with SCZ (7 female, 36.83 ± 8.32 years) with the DSM-IV-TR diagnosis of SCZ were recruited from outpatient units of the Charité-Universitätsmedizin Berlin. The psychiatric assessment was carried out by a senior psychiatrist at the recruiting institution. To minimize distorting effects of medication on the EEG, we did not recruit any participants who were taking benzodiazepines, lithium, or valproic acid. Details of medication can be seen in **Table 1**. Twenty-four education, handedness, gender-, and age-matched HC participants (8 female, 36.50 ± 8.47 years), who were screened for psychopathology with the German version of the Structured Clinical Interview for DSM-IV-TR Non-Patient Edition (SCID), were recruited from the general population (**Table 1**). All participants were tested with the Brief Assessment of Cognition in Schizophrenia (BACS) (31). Severity of symptoms was obtained by the Positive and Negative Syndrome Scale (PANSS) by trained clinicians (32). In accordance with a five-factor model, items were grouped into factors “positive,” “negative,” “depression,” “excitement,” and “disorganization” (33). All participants gave written informed consent, had normal hearing and normal or corrected to normal vision, and no record of neurological disorders. No participant met DSM-IV-TR criteria for alcohol or substance abuse. A random sample of about 40% of participants underwent a multidrug screening test. The study was performed in accordance with the Declaration of Helsinki and the ethics commission of the Charité-Universitätsmedizin Berlin approved the study.

**Table 1:** Descriptive data for schizophrenia (SCZ) and healthy control (HC). Demographic information, medication, Positive and Negative Syndrome Scale (PANSS), and Brief Assessment of Cognition in Schizophrenia (BACS).

	SCZ		HC		Statistics	
	Mean	SD	Mean	SD	*t*-values	*p*-values
Age (years)	36.83	8.32	36.50	8.47	.13	.86
Education (years)	10.78	1.65	11.08	1.60	−.64	.52
Daily cigarettes	5.87	5.01	3.04	3.69	2.21	.032*
Illness duration (years)	9.04	4.99	—	—	—	—
Chlorpromazine eq.	401.7	192.1	—	—	—	—
	***n***		***n***			
Gender (m/f)	16/7		16/8			
Handedness (r/l)	19/4		21/3			
Antipsychotic med.	23		—			
Haloperidol	1		—			
Amisulpride	6		—			
Clozapine	6		—			
Quetiapine	1		—			
Olanzapine	6		—			
Aripiprazole	4		—			
Risperidone	6		—			
Paliperidone	1		—			
Antidepressive med.	3		—			
Mirtazapine	1		—			
Escitalopram	2		—			
Paroxetine	1		—			
**BACS**	**Mean**	**SD**	**Mean**	**SD**	***t***	***p***
Verbal memory	42.65	12.82	49.92	11.46	−2.05	.046*
Digit	40.91	11.65	45.50	11.96	−.133	.190
Motor	43.04	11.32	51.67	9.42	−2.85	.007*
Fluency	44.83	11.81	49.79	13.48	−1.34	.187
Symbol coding	42.09	10.97	45.79	12.65	−1.07	.290
ToL	49.17	8.90	51.25	7.09	−.887	.380
Total score	244.0	38.73	273.2	36.45	−2.67	.011*
**PANSS**	**Mean**	**SD**	**Mean**	**SD**	***t***	***p***
Positive	10.00	1.88	—	—	—	—
Negative	15.17	2.44	—	—	—	—
Disorganized	7.57	1.78	—	—	—	—
Excited	8.17	1.07	—	—	—	—
Depressed	8.17	1.27	—	—	—	—

Statistics: two-tailed independent groups t-tests, *p < 0.05.

### EEG Recording

We recorded at least 5 min of eyes-open resting EEG data for each participant. During the recording, participants were asked to keep their gaze on a small fixation cross, which was shown on a display placed in front of them. Data were recorded using a 128-channel active EEG system (EasyCap, Herrsching, Germany), which included two EOG electrodes (online: 1,000 Hz sampling rate with a 0.016- to 250-Hz bandpass filter; offline: 49–51 Hz; fourth-order Butterworth notch filter, 125 Hz 24th-order FIR lowpass filter; downsampled to 500 Hz; 1 Hz 1,500th-order FIR highpass filter). To correct for EOG and ECG artifacts, independent component (IC) analyses were conducted (34). On average, 12.17 ± 5.46 ICs for SCZ and 13.42 ± 8.72 ICs for HC were rejected based on visual inspection (35). We used spherical interpolation to interpolate remaining noisy channels (SCZ = 10.61 ± 4.96 channels; HC = 9.25 ± 5.47 channels). Finally, we re-referenced continuous data to the average of all EEG electrodes, excluding remaining noisy data segments by visual inspection.

### LRTC Analysis

**Figure 1:**
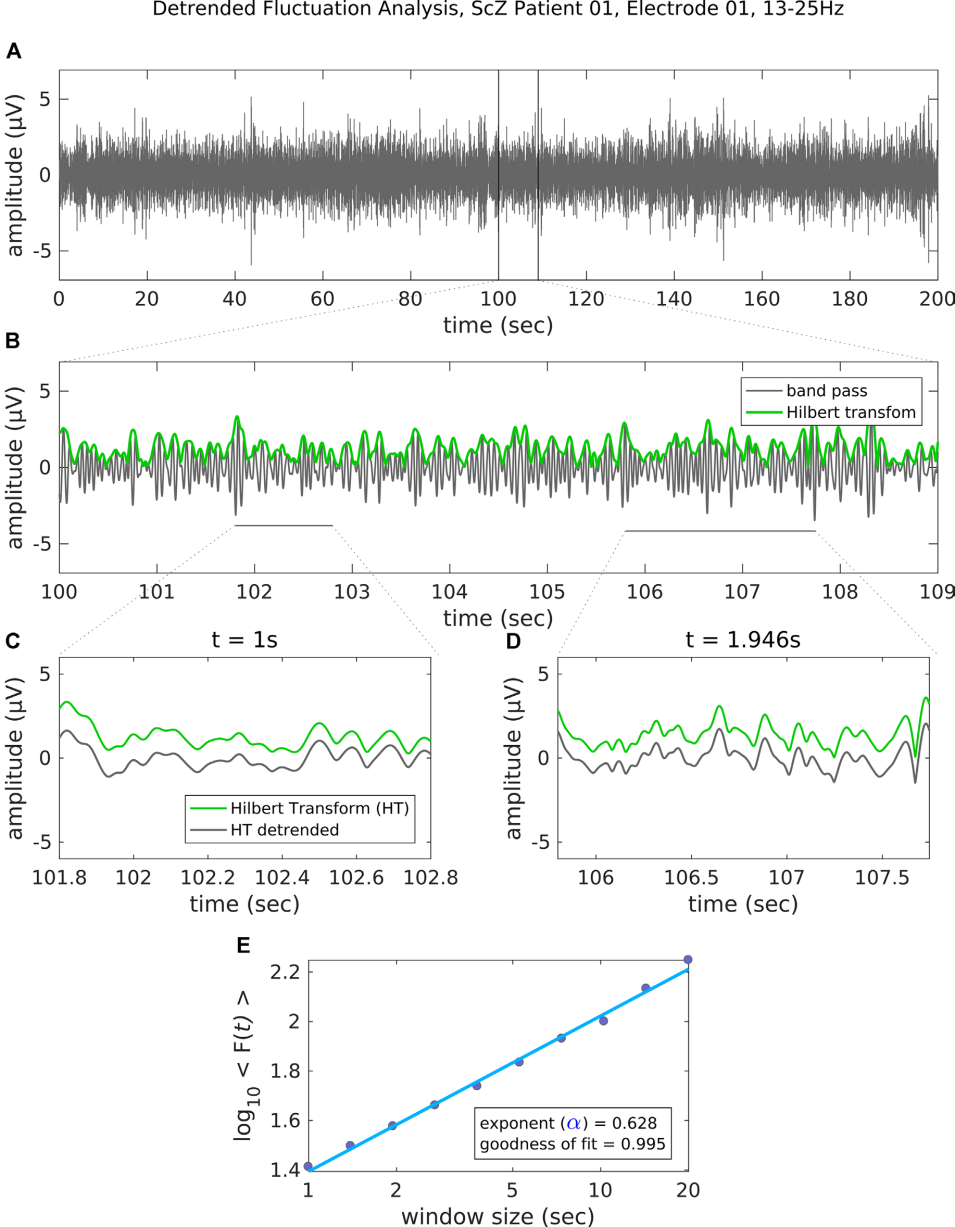
Steps of the detrended fluctuation analysis (DFA). **(A)** The band-pass filtered (13–25 Hz) EEG recording (channel 01 = Cz) of 200 s in a representative patient with schizophrenia. **(B)** An enlarged segment (9 s) of the band-pass filtered data with the corresponding amplitude envelope (Hilbert transformation, green) used for the estimation of the scaling exponent. **(C–D)** The amplitude envelope for two example window sizes before (green line) and after (gray line) detrending (i.e., removal of the linear trend). For each time window size, the fluctuation of the detrended signal is then estimated as the mean standard deviation of all identical sized segments **(E)** Results of the DFA. The scaling exponent is the slope of the log-log plot between fluctuation F and window size. An exponent 0.5 < α ≤ 1 indicates the presence of LRTC for up to 20 s. In this example case, the scaling exponent for the beta-band-oscillations in this channel is α = 0.628. Goodness of fit score is the *R* ^2^ value of the linear regression.

In the next step, the artifact-cleaned data were band-pass filtered using a second-order, two-pass Butterworth filter in the range 8 to 12 Hz for alpha and 13 to 25 Hz for beta oscillations. Next, we applied the Hilbert transformation to extract the amplitude envelope (36). The Hilbert-transformed signals were then analyzed using the detrended fluctuation analysis (DFA) (37, 38). DFA is a robust method for quantifying the long-range temporal correlations (LRTC; i.e., the scale-invariance) in the amplitude envelope of neuronal oscillations. It does this by estimating the power law scaling exponent describing the relation between amplitude fluctuation and the temporal window size (16, 37). It is formally expressed as: *F*(*s*) ∝ s^α^, with *F* corresponding to the average amplitude fluctuation (SD) for a specified window size *s*. A scaling exponent with values 0.5 < α ≤ 1 indicates the presence of LRTC, whereas α = 0.5 demonstrates that the data are completely uncorrelated. **Figure 1** depicts some basic steps of the DFA analysis in a representative case. We analyzed the LRTC in a time ranging from 1 s up to 20 s with 10 window sizes distributed equidistantly on a logarithmic scale. Although small window sizes might introduce a positive bias to the estimated scaling exponent (39), the bias effect would be quite small and equal for all channels and conditions. Therefore, a small window size of 1 s is not expected to affect the statistical comparisons (5, 40). The available segments of maximal length (20 s) after artifact correction were on average 12.5 (SD, 1.9) per subject, and there was no significant difference between the groups [12.7 (SD, 1.8) for SCZ; 12.4 (SD, 2.0) for HC; paired *t*-test, *t* (45) = −0.46, *p* = 0.65]. To control whether the amplitude of alpha and beta oscillations differed between people with SCZ and HC, we estimated the mean amplitude by averaging the values of the amplitude envelope across time, separately for each sensor and subject (21, 41).

### EEG Source Analysis

To visualize cortical sources of the LRTC in SCZ and HC groups, we projected LRTC values into source space. To this end, we combined sensor-level LRTC values of all participants and computed the covariance across all EEG channels. Common spatial filters for all participants were then computed from this covariance matrix using the FieldTrip implementation of sLoreta (42). Common spatial filters ascertain that source-space differences between groups are not due to differences in sensor-level data. Next, we computed the covariance across LRTC values for all EEG channels within each participant, and we projected LRTC values into source space using the previously computed common spatial filters, the MNI standard MRI, and boundary-element volume conductor model (BEM). The sLoreta-algorithm calculates the current density for a fixed set of dipoles based on a three-dimensional grid with 1-cm spacing computed from the aforementioned BEM. We set the spatial smoothing parameter lambda to 5%. We averaged source-level LRTC values within SCZ and HC and projected them onto the cortical surface of the MNI standard brain. For visualization purposes, projected LRTC values were masked by a common threshold of 70% of the between-groups maximum (**Figure 4**). This approach differs from the computation of LRTC on the source level (43) and does not permit statistical comparisons at the source level.

### Statistical Analysis

To analyze statistical differences in the oscillatory amplitude and the LRTC values at the sensor level, we performed non-parametric, cluster-based permutation test using the “FieldTrip” software [independent samples *t*-test, 1,000 iterations (44)]. The experimental cluster test statistic was evaluated against the Monte-Carlo permutation distribution to test the null hypothesis of no differences between the conditions (HC–SCZ). We set the threshold to control for family-wise error (FWE) to *p* = 0.025 (two-sided test) and performed a cluster-based contrast HC vs. SCZ for alpha and beta LRTC values. The initial cluster-forming threshold was set to *p* = 0.05. We employed Spearman-ranked correlations to test the relation of LRTC outcomes with PANSS and BACS scores.

## Results

**Figure 2:**
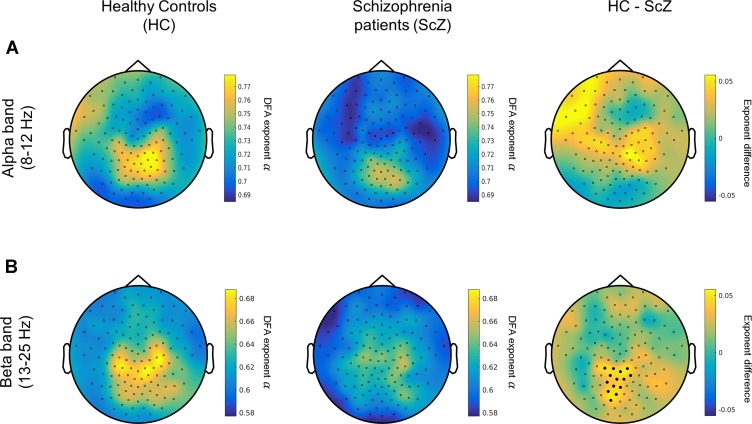
People with SCZ display weaker long-range temporal correlations (LRTC) in the beta oscillations (13–25 Hz). **(A)** Left and middle panels depict the topographical distribution of alpha-band (8–12 Hz) LRTC values for healthy control (HC) (left) and people with schizophrenia (SCZ) (middle). The difference HC–SCZ is displayed on the right panel. The cluster analysis revealed no statistically significant alpha-band LRTC differences between the two groups. **(B)** The topographical distribution of the beta-band LRTC values for HC (left) and SCZ (middle) as well as the beta LRTC difference between the two groups (HC–SCZ; right). A cluster-based permutation test revealed that HC had significantly stronger beta-LRTC over central-posterior electrodes compared with people with SCZ. Black dots denote the channels contributing to the significant cluster.

To assess the differences in the degree of temporal dependencies in the EEG signals between people with SCZ and HCs, we contrasted the scaling exponent values at the sensor level, separately for alpha and beta frequency bands (**Figure 2**). A cluster-based analysis HC vs. SCZ for the beta-band LRTC values demonstrated a significant cluster with stronger beta LRTC values for HC over parieto-occipital electrodes (*p* < 0.025, **Figure 2**). This finding indicates that people with SCZ had weaker beta-band temporal correlations compared with healthy individuals. The analysis for the alpha-band LRTC values revealed no significant group differences.

**Figure 3:**
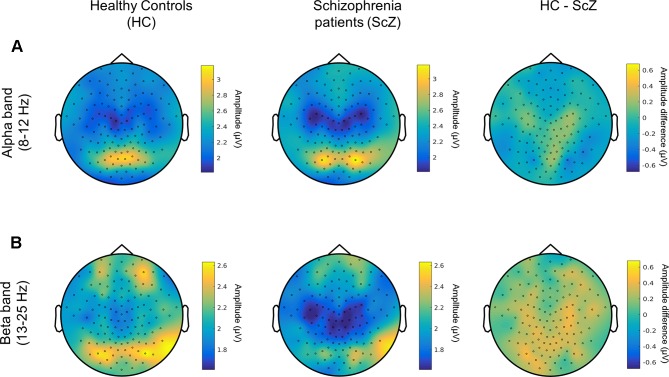
Amplitude of alpha and beta neuronal oscillations in HC and SCZ **(A)** Topographical distribution of the amplitude of alpha oscillations (8–12 Hz) is displayed for HC (left) and people with SCZ (middle). The difference between the two groups is also shown (HC-SCZ; right). A cluster-based permutation test showed that there is no significant difference in the amplitude of alpha oscillations between the two groups. **(B)** Topographical distribution of the amplitude of beta band oscillations (13-25 Hz). The cluster-based analysis showed that the amplitude of beta oscillations did not significantly differ between HC and SCZ.

**Figure 4:**
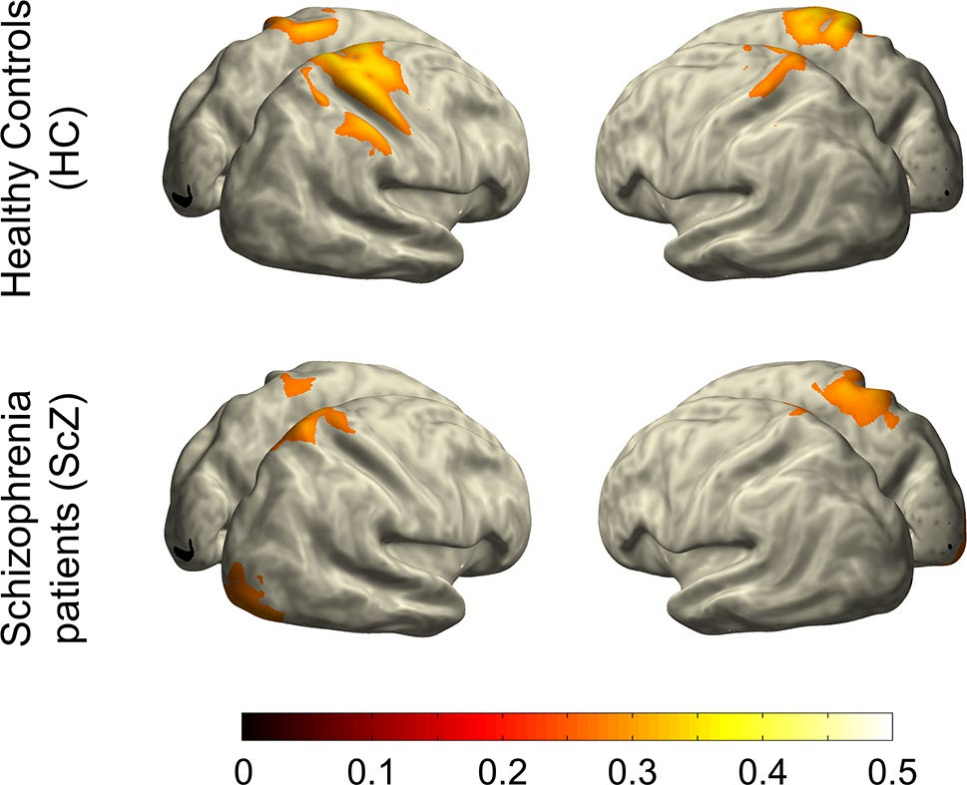
Projection of beta-band LRTC into source space. This illustration indicates that the weakened long-range temporal correlations of beta-band oscillatory activity in people with SCZ relative to HCs is primarily localized in bilateral posterior regions of the cortex.

A control analysis using the same cluster-based approach demonstrated that the mean amplitude of alpha and beta oscillations (i.e., the average of amplitude envelope values across time) between HC and SCZ was not significantly different, indicating that the observed beta-band LRTC effect was not affected by any amplitude differences between the two groups (**Figure 3**). This finding is in agreement with previous research (21). The projection of the beta-band DFA exponents in source space suggests that the altered beta-band LRTC in people with SCZ has a bilateral posterior topography (**Figure 4**).

The results show no significant correlation with antipsychotic medication levels, operationalized as chlorpromazine equivalent (*r* = −0.192, *p* = 0.381; 45). This indicates that the LRTC differences between people with SCZ and HCs were not the result of medication differences. Since long-term medication could have an effect on brain architecture, and therefore EEG, we performed an additional multiple regression analysis of illness duration and chlorpromazine equivalent upon beta band LRTC. The model was not significant, predicting only 8% of the variance (R^2^ = 0.08, *F*[3,19] = 0.56, *p* = 0.65). Neither of the individual predictors nor their interaction predicted LRTC in the beta band (chlorpromazine equivalent [β = 0.40, *p* = 0.49], illness duration (β = 0.92, *p* = 0.26), and chlorpromazine equivalent × illness duration [β = −1.15, *p* = 0.29]). Moreover, there were no significant correlations between beta-band LRTC values and the dimensions of the PANSS (32) (*N* = 23; positive, *r* = 0.01; negative, *r* = −0.23; disorganized, *r* = −0.001; excited, *r* = 0.17; depressed, *r* = −0.09; all *p* values > 0.30). Similarly, there were no significant correlations between beta-band LRTC values and the five dimensions of the (BACS) (31) (*N* = 23; verbal memory, *r* = 0.08; digit sequencing, *r* = 0.12; token motor, *r* = 0.11; verbal fluency, *r* = 0.23; symbol coding, *r* = 0.03; tower of London, *r* = −0.05; all *p* values > 0.2).

## Discussion

In this study, we examined oscillatory temporal dynamics in individuals with SCZ and HCs. We found diminished LRTC in the beta frequency band in people with SCZ relative to HCs. Source projection of the reduced beta-band LRTC in the SCZ group indicated an involvement of bilateral posterior brain regions. Unlike Nikulin et al. (21), we did not find an attenuation of LRTC in people with SCZ for the alpha frequency band. Moreover, there were no significant correlations between LRTC and either clinical or cognitive parameters.

Source projection with high-density EEG located the reduction in beta-band LRTC to bilateral posterior regions. These reductions may reflect specific instability of networks in sensorimotor regions. Parietal beta oscillations likely play a role in maintaining network memory (24). Moreover, beta and gamma activity interact in complex ways (25, 46). A model from Kopell et al. (25) shows that parietal beta oscillations, originating in deeper cortical layers, help to maintain gamma networks, which are otherwise stimulus-dependent, transient, and competitive. Since SCZ patients often show posterior gamma reductions in task-related processing (1), the reduced beta-band LRTC in our study and in the study by Nikulin et al. (21) could play a role in this instability. One way that future experiments could test this is by examining parietal beta-band LRTC and gamma band power in task-related paradigms, where SCZ have shown behavioral deficits. Since LRTC analyses have already been extended beyond resting-state measures into task-based EEG measures in healthy participants [e.g., sensorimotor tasks (47) and audiovisual threshold (43)], the connection between parietal beta LRTC and gamma power could be tested on already existing data.

From the perspective of neurochemistry, it is likely that dopamine (DA), which is important in stabilizing neural networks (17, 48), plays a crucial role for SCZ psychopathology (49). Moreover, the temporal dynamics of beta oscillations, as reflected in the LRTC, could be sensitive to the DA levels in neural networks. For example, the administration of DA precursor Levodopa for the treatment of Parkinson patients resulted in an increase of beta-band LRTC in the subthalamic nucleus (50). Nevertheless, the putative role of the dopaminergic system for the beta-band LRTC deficits in SCZ patients is speculative at this point. Future research using combined EEG and positron emission tomography could examine the possible connections between DA and aberrant beta-band LRTC in people with SCZ.

In contrast to Nikulin et al. (21), who found significantly reduced parietal-occipital LRTC in the alpha-band, we did not find group differences in alpha-band LRTC. In Nikulin et al.’s (21) study, participants closed their eyes, while they were asked after each minute to briefly open them to prevent sleep. In our study, participants had their eyes open throughout the entire EEG recording session. This was to ensure consistent visual input, as well as to better control the vigilance level across groups. Notably, a previous study in healthy participants did not find differences in alpha-band LRTC in eyes-open vs. eyes-closed conditions (16). Nevertheless, it is possible that the eyes-closed vs. eyes-open conditions could have changed the LRTC in the alpha band in SCZ. However, this manipulation was not carried out in our experiment and would have to be tested in a future experiment.

Posterior alpha power is stronger in eyes-closed compared with eyes-open resting state conditions (26, 27). Therefore, one could argue that increased power in an eyes-closed state might give it more statistical sensitivity for the detection of alpha band LRTC differences. However, previous studies (21, 51), as well as our own beta-band results, do not show a link between LRTC differences and power differences. Indeed, Linkenkaer-Hansen et al. (51) suggest that the lack of relation between LRTC and power indicates that they might be driven by different biophysical mechanisms. This is also supported by a study showing an opposite effect of Levodopa administration on beta band LRTC (increase) and power (decrease) in patients with Parkinson disease (50).

Our study contrasted people with SCZ and HCs and treated antipsychotic medication as a potential confound variable. Medication is known to have effects on the EEG signal (52, 53); however, results specifically testing this on LRTC yield contrasting findings. For example, Gärtner et al. (41) tested differences between medicated and non-medicated depressive patients and found no differences. On the other hand, Hohlefeld et al. (50) found that beta LRTC in LFP recordings from the subthalamic nucleus in patients with Parkinson’s were increased after Levodopa administration. In our data, there was no significant correlation between the chlorpromazine equivalence metric and LRTC, making it less likely that the group differences are attributable to medication. Since medication could have a long-term effect on brain architecture in SCZ (53), we additionally tested illness duration together with medication in a regression model, but found no effects of these factors on beta band LRTC. It is, nevertheless, possible that some medication effect would be detectable in a larger sample. This requires further testing. For example, if the posterior beta-band activity is related to DA-mediated cortico-striatal activity, future studies could examine causal effects of antipsychotic medication as an experimental variable, for example contrasting first episode SCZ before and after treatment, as done in Sarpal et al. (54). In terms of both clinical subsymptoms and cognitive performance, participants with SCZ were relatively high functioning. Although they could be clearly clinically distinguished from the control group, the range of variance in symptomatology might not have been wide enough to make it sensitive to potential relationships between beta-band LRTC and subsymptoms. This could explain the absence of relationships between LRTC alterations in SCZ and clinical parameters, i.e., positive and negative symptoms, as measured by the PANSS, as well as cognitive parameters, measured by the BACS. Despite these limitations, by replicating the diminished beta-band LRTC for SCZ in (i) a larger participant sample, (ii) with a higher number of EEG electrodes, and (iii) using rigorous statistical analytic approaches, our study advances the understanding of cortical processing alterations in the temporal domain during rest in SCZ. In the present study, source analyses were performed for illustration purposes only. The precise estimation and statistical analysis of the LRTC at the source level requires further analyses that were beyond the scope of the present study, partly as the data quality does not allow an adequate control for false positives. A more elaborated source-space analysis of the LRTC would require source reconstruction of EEG data and subsequent estimation of the LRTC on the source-space time series. In addition, source reconstruction would benefit from access to the individual electrode positions and MRI scans, which were not obtained in the current study [see Ref. (43), for an example of rigorous source-level LRTC analyses].

Taken together, our study strengthens the assumption that alterations in beta-band LRTC serve as a neurophysiological marker for altered temporal stability within cortical networks in SCZ. Among others, beta-band oscillations have been related to motor processing and top-down modulation. The outcome of our source space projection indicates an involvement of bilateral posterior brain regions, as putative sources of the aberrant beta-band LRTC in SCZ. Furthermore, the absence of alpha-band LRTC alterations during rest suggests that differences in LRTC are most robust in the beta-band. The current study confirms the utility of LRTC for investigating dysconnection in SCZ and suggests that aberrant beta-band LRTC reflects altered temporal dynamics in the cortical networks of people with SCZ.

## Ethics Statement

The authors assert that all procedures contributing to this work comply with the ethical standards of the relevant national and institutional committees on human experimentation and with the Helsinki Declaration of 1975, as revised in 2008. The ethics committee at the Charité-Universitätsmedizin Berlin, approved the experimental procedures.

## Author Contributions

Study Design: JK, DS, Data Collection: JK, Data Analysis: GM, JK, JM, DS; Manuscript preparation: JM, GM, JK, AH, DS.

## Funding

This work was supported by grants from the European Union (ERC-2010-StG-20091209 to DS) and the German Research Foundation (KE1828/4-1 to JK, and SE1859/4-1 to DS).

## Conflict of Interest Statement

The authors declare that the research was conducted in the absence of any commercial or financial relationships that could be construed as a potential conflict of interest.
